# Genetic and Morphological Evidence From a Group of Rare African Free‐Tailed Bats Reveals a New Subgenus Within *Mops*

**DOI:** 10.1002/ece3.71288

**Published:** 2025-05-14

**Authors:** Laura Torrent, Inazio Garin, Joxerra Aihartza, Aline Méndez‐Rodríguez, Esther Abeme Nguema Alene, Javier Juste

**Affiliations:** ^1^ BiBio (Biodiversity and Bioindicators Research Group) Natural Sciences Museum of Granollers Granollers Spain; ^2^ CIBIO‐InBIO, Research Centre in Biodiversity and Genetic Resources University of Porto Vairão Portugal; ^3^ Biodiversity Initiative Houghton Michigan USA; ^4^ Department of Zoology and Animal Cell Biology UPV/EHU University of the Basque Country Leioa Spain; ^5^ Universidad Autónoma Metropolitana Lerma México DF Mexico; ^6^ National Institute for Forest Development and Management of the Protected Areas Bata Equatorial Guinea; ^7^ Department of Evolutionary Ecology Estación Biológica de Doñana (CSIC) Sevilla Spain; ^8^ Epidemiology and Public Health CIBERESP Madrid Spain

**Keywords:** *Chaerephon*, Congo rainforest, Equatorial Guinea, *Mops*, phylogenetics, taxonomy

## Abstract

Recent surveys in the Congolian rainforest have significantly improved the quantity and quality of material available to rigorously assess bat diversity (Order Chiroptera) in this biodiversity hotspot. However, the paucity of data on free‐tailed bats in this region is hindering our ability to resolve the actual number of species present in Central Africa. During a recent expedition to continental Equatorial Guinea, a single free‐tailed bat was captured in a patch of primary continental rainforest. This bat exhibited unique external and cranial characteristics, strongly suggesting it to be a male of *Mops tomensis*, which was originally described as an endemic species from the oceanic island São Tomé in the Gulf of Guinea. The original description of this species was based on just three females collected 30 years ago. DNA from a paratype of *M. tomensis* fully supported that the newly captured specimen represents the first documented male of the species and extends its known distribution to mainland Africa. Furthermore, genetic analyses revealed a high divergence between these two individuals of *M. tomensis* and other species of the genus, and both clustered within a distinct, well‐supported clade. With this genetic evidence, along with the unique morphological features of *M. tomensis*—like the prominent interaural lobe that is shared only with one other enigmatic free‐tailed bat from the Democratic Republic of the Congo (*Mops gallagheri*)—we describe a new subgenus within *Mops*, as previously suggested for *M. gallagheri*. These findings demonstrate that even small‐scale sampling can yield significant discoveries and expand the known distribution of rare bats in the Congolian rainforest. This underscores the urgent need to prioritise biodiversity studies in the region to fully assess its richness and implement effective conservation measures.

## Graphical abstract

Recent surveys in the Congolian rainforest have improved knowledge of bat diversity, but data on free‐tailed bats remain scarce. A male *Mops tomensis*, previously known only from São Tomé, was captured in Equatorial Guinea, extending its range to mainland Africa. Genetic analyses confirmed its identity and revealed a distinct lineage, leading to the description of a new *Mops* subgenus. These findings highlight the importance of biodiversity surveys for uncovering cryptic species and informing conservation efforts.

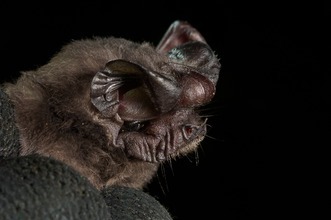

## 1 Introduction

The rising number of species described in the Order Chiroptera over the last two decades has prompted the term ‘Age of Discovery for Bats’ (Tsang et al. 2016). The family Molossidae, or free‐tailed bats, are open‐flying bats recognised by their exclusive tail and wing morphologies and present a mainly tropical distribution worldwide (Taylor et al. 2019). It is commonly accepted that the family diverged from other related bats (Vespertilionidae and Miniopteridae) in the Palaeocene, at least 50 million years ago (Miller‐Butterworth et al. 2007). The family consists of typically aerial insectivorous bats and comprises at present 126 species grouped in 22 genera (Taylor et al. 2019). In Africa, there are 64 recognised species (Van Cakenberghe and Seamark 2024), several of which were described in the past 15 years, such as *Mops bakarii* (Stanley 2008), *Mormopterus francoismoutoui* (Goodman et al. 2008), *Mops atsinanana* (Goodman et al. 2010) and *Otomops harrisoni* (Ralph et al. 2015). Several of the African molossid bats—mainly forest‐dwellers—are still known from a single locality or by a handful of specimens collected in the twentieth century and scattered across museum collections (e.g., *Mops congicus* , *M. tomensis*, *M. gallagheri* , *M. niangare* and *M. petersoni* ).

The systematics and evolutionary relationships within the Molossidae family remain poorly resolved. It represents the last major bat family still in need of a comprehensive systematic and taxonomic appraisal. Efforts to clarify the systematics and evolution of free‐tailed bats have been hindered by a relatively conservative morphology, combined with a limited representation of genetic data in terms of sampled species and the number of markers. These challenges are further compounded by frequent discrepancies between morphological and molecular approaches (Freeman 1981; Legendre 1984; Lamb et al. 2011; Ammerman et al. 2012; Napier 2013; Shi and Rabosky 2015; Gregorin and Cirranello 2016). In a seminal comparative study based on comprehensive morphological analyses, Freeman (1981) divided around 80 free‐tailed species into two groups: the *Mormopterus*‐like and the *Tadarida*‐like bats. Later, Legendre (1984) subdivided the family into three subfamilies based specifically on dental morphology: Cheiromelinae, Molossinae and Tadaridinae.

From a genetic perspective, Miller‐Butterworth et al. (2007) propose that an early split gave rise to two subfamilies: Tomopeatinae, a relict and monospecific lineage found in the deserts of Perú (Sudman et al. 1994) and Molossinae, which comprises all the remaining free‐tailed bats. Molossinae was further divided into Old World and New World lineages, which split around 29 million years ago (Ammerman et al. 2012). Unfortunately, relationships among genera and species within the subfamily Molossinae are still unclear despite attempts using both nuclear and mitochondrial DNA markers (e.g., Lamb et al. 2011; Ammerman et al. 2012; Napier 2013). Morphological and genetic conservativism at supra‐specific levels complicates phylogenetic analyses and species recognition. This is shown by the recent revision of the entangled group of Neotropical free‐tailed bats included within the genus *Molossus* (Loureiro et al. 2019, 2020) that implied the clump of several traditional taxa. Another example of a long‐lasting unsolved muddle relates to the taxa *Chaerephon*, *Mops* and *Tadarida*, mainly Old World free‐tailed bats. All three taxa were described in the nineteenth century and Freeman (1981) grouped them within the *Tadarida*‐like group due to their external and cranial similarities, but their classification has been debated. Legendre (1984) and Happold and Happold (2013) treated *Chaerephon* and *Mops* as subgenera of *Tadarida*. In contrast, Freeman (1981) and Koopman (1993) arranged them as independent genera. This distinction was based mainly on differences in the ears (whether joined or separated) and the presence or absence of wrinkles on the upper lip. More recently, in an exhaustive cladistic analysis of morphological characters, Gregorin and Cirranello (2016) found weak support for a grouping of *Chaerephon* and *Mops* but confirmed that they are distinct from *Tadarida*. Molecular analyses have evidenced that both *Chaerephon* and *Mops* are paraphyletic as defined at present and also suggest that ‘strictly speaking’ *Chaerephon* should be included within the genus *Mops* and both considered distinct from *Tadarida* (Lamb et al. 2011; Ammerman et al. 2012; Napier 2013). It seems clear that the much needed redefinition of these taxa and the clarification of their systematic and phylogenetic relationships will require further analyses including the information of more molecular markers and a much larger representation of taxa. Provisionally, it seems appropriate to follow the available phylogenetic hypotheses (see Napier 2013) that suggest the inclusion of *Chaerephon* as a subgenus within *Mops* until this controversy is finally settled.

The morphological differentiation within Molossidae is further complicated by the notable sexual dimorphism presented by the family (Freeman 1981). Unlike some bat families, in which sexual dimorphism often favours larger females to support reproductive and flight efficiency (Ralls 1976; Meyers 1978; Lindenfors et al. 2007), molossid bats typically exhibit male‐biased dimorphism (Freeman 1981; Taylor et al. 2019). In addition to the size differences, male molossids display marked secondary sexual characters, such as gular glands, brighter pelage colouration and distinctive craniodental features, such as variations in canines, incisors and lambdoidal crests (Freeman 1981; Racey 2009). These morphological differences between sexes have been useful to tell apart related species (Juste and Ibáñez 1993) but they can also hinder accurate identification of specimens in museum collections. Indeed, it is difficult to tell apart female specimens of *Mops spurrelli* and *Mops nanulus* , as both present highly similar external and craniodental morphology (Happold 2013a). This challenge is particularly pronounced in taxa with limited availability of specimens in collections. For example, *Mops* (*Chaerephon*) *gallagheri* is represented by only a male specimen (Harrison 1975), while *Mops* (*Chaerephon*) *tomensis* is known solely from three female specimens (Juste and Ibáñez 1993).

Studying a collection of bats made in forests of the Congo River in central Democratic Republic of the Congo, Harrison (1975) described a small molossid bat presenting outstanding unique external and cranial features. The new species, named as *Tadarida gallagheri* in honour of its collector, exhibited a deep interaural pocket, encasing a tuft of hairs similar to other free‐tailed bats (e.g., *Mops chapini*). However, in this case, ‘*gallagheri*’ presented an anterior extension in the band of skin connecting the ears, forming a bulbous protrusion that overhung in front of the muzzle (Harrison 1975). In addition, the skull exhibited unique large lateral nasal inflations that gave an ‘extremely unusual’ truncated shape to the rostrum. Harrison (1975) stated that these bizarre characteristics were sufficient to demand consideration of generic status. However, he was finally refrained from describing it because ‘*gallagheri*’ was similar in other characteristics to other *Chaerephon* species and a single specimen was thought to provide too weak evidence to justify the establishment of a new genus. Twenty years later, another forest free‐tailed species was found in the remote oceanic island of São Tomé and described as *Chaerephon tomensis* from a type series of three female specimens (Juste and Ibáñez 1993). These specimens exhibited again the unusual interaural band and rostrum inflations, although less pronounced than in *M*. (*Ch*.) *gallagheri*.

**FIGURE 1:**
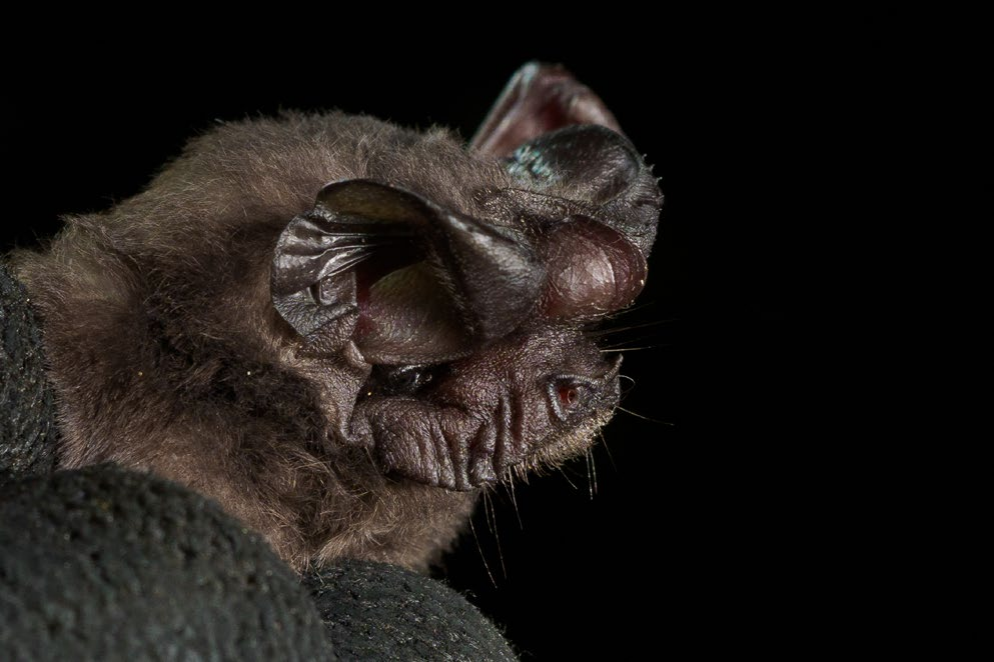
Portrait of a *Mops tomensis* male (EBD 34943M) captured in Piedra de Nzas National Monument, mainland Equatorial Guinea. Photo credit: LT.

In 2023, we carried out an expedition in the continental region of Equatorial Guinea, Central Equatorial Africa and collected a small molossid bat captured in a canopy mist‐net set in the primary forest of ‘Piedra de Nzas’ National Monument (Figure 1). Surprisingly, this bat was exhibiting again the unusual unique morphological characteristics shared by *gallagheri* and *tomensis*. This new bat supports the idea of describing a new group of African free‐tailed bats as Harrison (1975) had anticipated. Here we describe this taxon as a new subgenus tentatively placed within the genus *Mops*. We also present detailed comparisons of these unique morphological structures among the species of the new group. Molecular analyses enabled the taxonomic identification of the mainland free‐tailed bat and provided insights into its phylogenetic relationships with other African species.

## 2 Materials and Methods

### 2.1 Study Area

**FIGURE 2:**
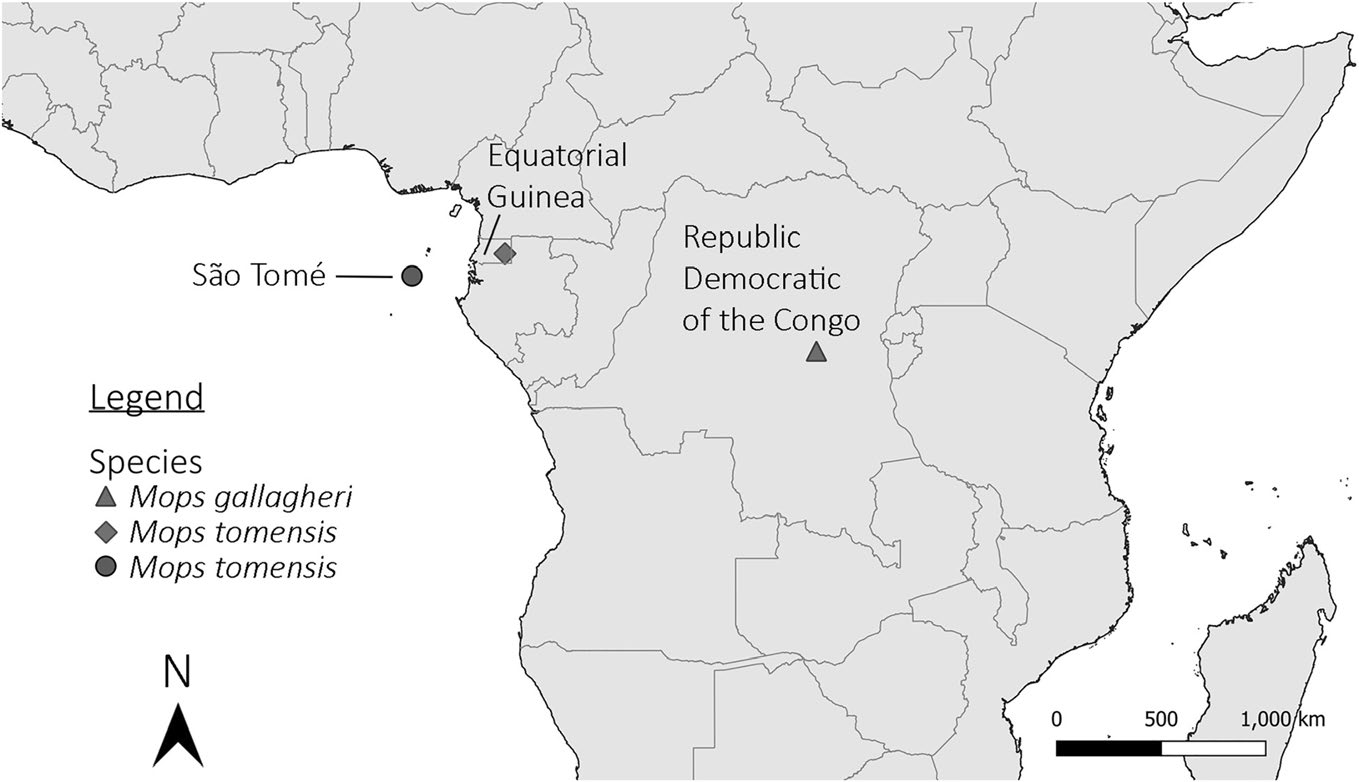
Type localities of *Mops tomensis* (lat. 0.400000, long. 6.616667) from São Tomé Island (circle) and *Mops gallagheri* (lat. −3.166667, long. 25.816667) from Kindu, Kivu region, Democratic Republic of the Congo (triangle). Locality for *Mops tomensis* (latitude 1.459300, longitude 11.030300) from Piedra de Nzas National Monument, mainland Equatorial Guinea (diamond).

Equatorial Guinea, with an overall land surface of 28,051 km^2^, is located in the Gulf of Guinea and consists of two main regions: the Atlantic islands of Bioko (2017 km^2^), Annobón (17 km^2^) and Corisco (15 km^2^) on the one hand, and the mainland region (26,017 km^2^) on the African mainland on the other (Rosas 2022) (Figure 2). Equatorial Guinea has a typically hot and humid equatorial climate with two seasons driven by rainfall regime, which significantly varies between regions. On Bioko Island, there is only one rainy season that lasts from March to October, with the dry season extending from November to February. Mean accumulated precipitation varies significantly across seasons and regions of the island (Rosas 2022). Meanwhile, in the mainland region, there are two marked rainy seasons: one from September to December and another from March to May; between these are two dry seasons: from December to February and from July to August. The average annual rainfall is 1800–3800 mm. The average temperature in the country is 25°C with oscillations of no more than 5°C all year round. *Source:* Grupo de Paleoantropología (MNCN‐CSIC) (Rosas 2022).

### 2.2 Molecular Analyses

The total genomic DNA of the new specimen collected in Equatorial Guinea was extracted from tissue samples preserved in 70% ethanol after isopropanol precipitation with saline purification, following Gemmell and Akiyama (1996). Two mitochondrial (mDNA) and one nuclear (nDNA) gene fragments were amplified by PCR. The molecular markers were selected based on previous studies on African molossids and included fragments of the mDNA cytochrome b (Cytb) and dehydrogenase subunit 1 (NADH1), and a nDNA fragment of the recombination activating gene 2 (RAG2). These fragments were amplified using the primers and conditions described in Table A1 in Appendix A. The amplification products were sequenced in the forward direction on an ABI 3100 (PE Biosystems, Warrington, UK) equipment. The sequences were manually edited and aligned using the Clustal Muscle algorithm implemented in Geneious software (Kearse et al. 2012). The resulting newly generated sequences from the Equatoguinean specimen were uploaded to GenBank (Tables A2 and A3 in Appendix A).

We followed a slightly modified extraction protocol (Campos and Gilbert 2012) with a paratype of *M*. (*Ch*.) *tomensis* (EBD 17371M) from São Tomé Island housed at the Doñana Biological Station (EBD‐CSIC) mammal collection. The paratype was immersed in formalin during its preservation process. DNA extracts were highly degraded, with the majority of fragments showing low molecular weight. The amplification of the fragment of Cytb from this degraded DNA required the design of a specific set of primers every 250 bp. The DNA of the specimen from Piedra de Nzas was used to design primer pairs to amplify homologous fragments of the formalin‐preserved paratype of *M*. (*Ch*.) *tomensis* (EBD 17371M).

To analyse evolutionary relationships, phylogenetic reconstructions were obtained using the longest possible fragment of the Cytb and also using the concatenated sequences of all three markers (Cytb, NADH1 and RAG2). Hypotheses were constructed under Bayesian inference (BI) after running 10,000,000 generations, with five Markov chains, and discarding the first 25% of trees as burn‐in, using the software MrBayes v.3.2 (Ronquist et al. 2012) on CIPRES Science Gateway v3.1 (Miller et al. 2010). Besides, evolutionary hypotheses were also obtained under maximum likelihood (ML) and maximum parsimony (MP) criteria. The analyses used heuristic search and node support was obtained after 1000 bootstrap replicates using the software PAUP* v.4.0b10 (Swofford 2002). The evolutionary model that better fitted the data for each data set was estimated with the software jModeltest v. 2.1.6 (Darriba et al. 2012) using the Bayesian information criterion (BIC). The species used in the phylogenetic trees were selected according to (1) their similarity index using nucleotide‐nucleotide standard BLAST (Altschul et al. 1990); and (2) their availability in the GenBank database (Tables A2 and A3 in Appendix A). MEGA v. 11.0.13 (Tamura et al. 2021) was used to estimate levels of genetic differentiation between species for Cytb according to a Kimura 2‐parameter (K2P) and *p*‐distances. Both estimates of genetic distance have been used in other studies (e.g., Goodman et al. 2008; Lamb et al. 2011; Moras et al. 2016) which is helpful for comparisons.

### 2.3 Cranial and Dental Morphometric Analyses

The free‐tailed bat from Piedra de Nzas was compared morphologically with the three specimens from São Tomé Island (*M*. (*Ch*.) *tomensis—*EBD 18256M, EBD 17734M, EBD 17371M), housed at EBD‐CSIC; the single specimen of *M*. (*Ch*.) *gallagheri* (NHM 1976.207) from the Democratic Republic of the Congo and a specimen of *M*. (*Ch*.) *chapini* (NHM 1937.12.8.25) from Zambia, both housed at the Natural History Museum of London (formerly The British Museum of Natural History) (BMNH). We measured three external and 12 craniodental characters with a digital calliper (to the nearest 0.1 mm for external and 0.01 mm for skull characters) following Happold and Happold (2013) nomenclature. Craniodental characters were measured under a stereo microscope. The morphological traits (in mm and g) and their abbreviations are as follows: forearm length (FA); body weight (W); total length of the body (TL); greatest length of skull without incisors (GLS); greatest height of skull (GHS); interorbital width (IOW); condylobasal length (CbL); mastoid width (MW); zygomatic width (ZW); palatal length (pal), after Kearney and Seamark (2005); palatal length (PL) after Csorba et al. (2003); width across upper molars (M^3^–M^3^); width across upper canines (C–C); width across upper incisors (I–I); upper toothrow measured from most anterior part of canine to most posterior part of M^3^ (C–M^3^).

## 3 Results

### 3.1 Phylogenetic Analyses

**FIGURE 3:**
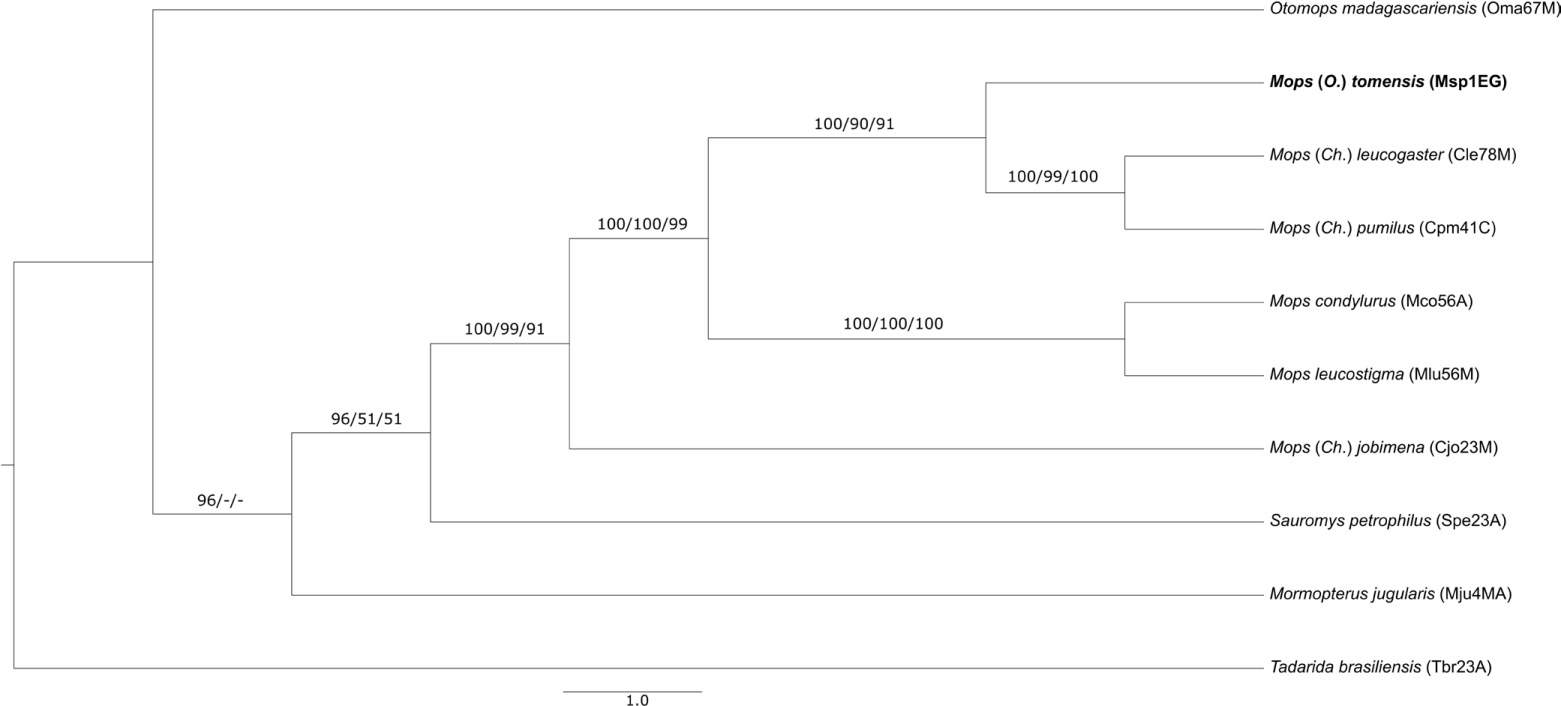
Phylogenetic reconstruction based on the concatenation of Cytb (325 bp), NADH1 (718 bp), and RAG2 (617 bp), with the support for each branch based on Bayesian posterior probability (BI)/bootstrap values of maximum likelihood (ML)/and bootstrap values of maximum parsimony (MP). See Table A3 in Appendix A for a complete list of the specimens used and the main text for details.

**FIGURE 4:**
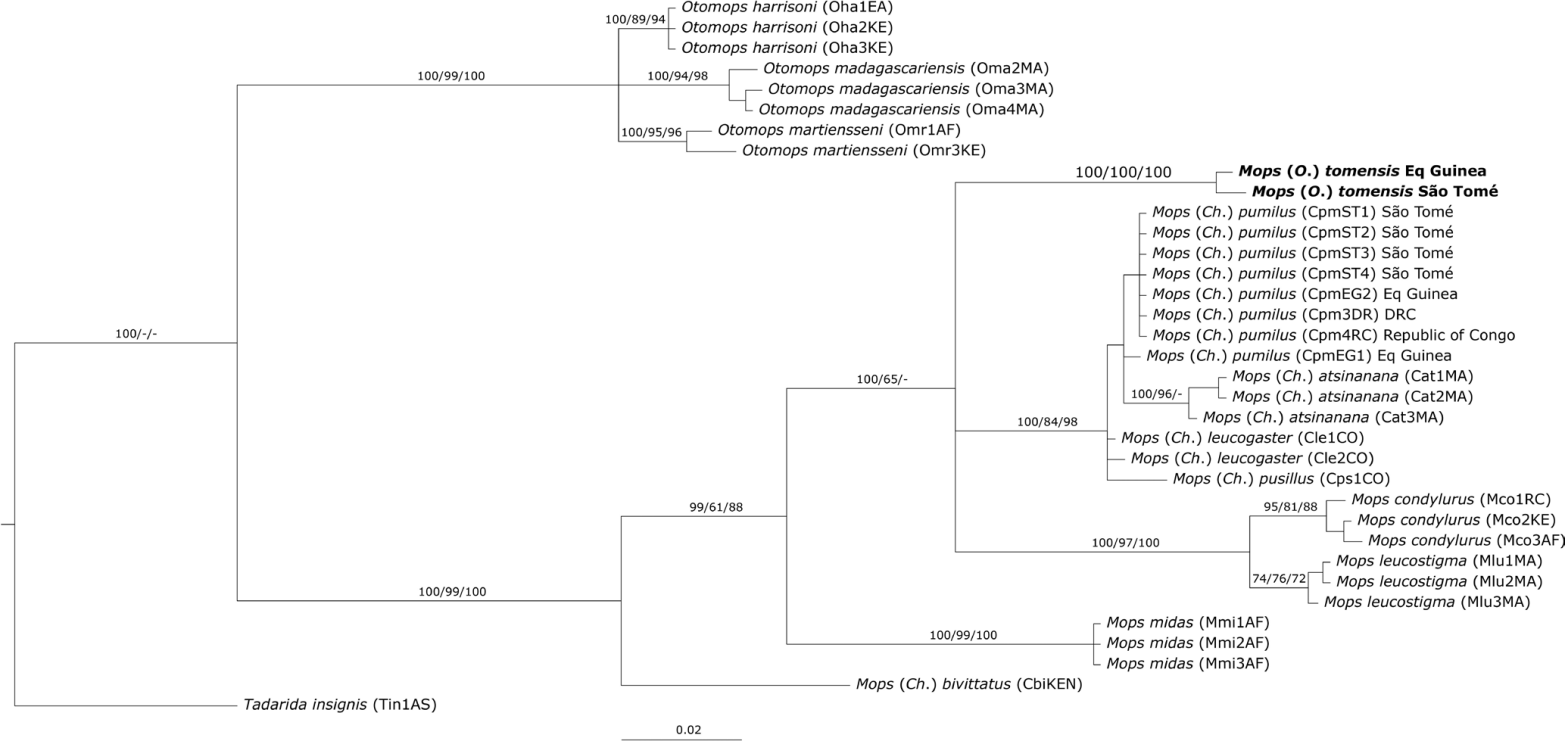
Phylogenetic reconstruction based on the mitochondrial gene Cytb (623 bp) with the support for each branch based on Bayesian posterior probability (BI)/bootstrap values of maximum likelihood (ML)/bootstrap values of maximum parsimony (MP). See Table A2 in Appendix A for a complete list of the specimens used and the main text for details.

We combined eight sequences generated in this study with 54 sequences downloaded from GenBank (Tables A2 and A3 in Appendix A). The length of the available sequences in GenBank constrained the length of Cytb fragments in each molecular analysis to 623 and 326 bp, respectively. Due to the low variability of RAG2, its topology lacked resolution, leaving most of the nodes unresolved and making separate mtDNA and nDNA trees uninformative. For the sole Cytb based analysis, 12 free‐tailed species were used in the phylogeny. Moreover, *Tadarida insignis* was used as an outgroup, and the evolutionary model selected was GTR + I + F. The concatenated analysis [Cytb (325 bp), NADH1 (718 bp) and RAG2 (617 bp)] was performed using nine free‐tailed species and *Tadarida brasiliensis* as an outgroup. Moreover, the selected evolutionary models, estimated by jModeltest, were TIM2ef + G for Cytb, TIM2 + I for NADH1 and TIM3 + G for RAG2. In both phylogenies (Cytb and concatenated), *Chaerephon* has a polytomy and *Mops* appears ancestral to *Chaerephon* (Figures 3 and 4). A clade comprising three species of *Otomops* is strongly supported in the Cytb phylogeny (Figure 4). The concatenated analysis found strong support, with Bayesian posterior probability (BI) estimates > 95, for *Mormopterus* being basal to *Sauromys* and the latter to *Mops* (Figure 3).

In all reconstructions, the free‐tailed bat from Piedra de Nzas joined *M*. (*Ch*.) *tomensis* in a well‐supported clade, confirming its species identity as *M*. (*Ch*.) *tomensis*. The BI based on the concatenated sequences yielded slightly more defined relationships than that based only on the Cytb (Figures 3 and 4). In fact, the topology based on the concatenated sequences clustered *M*. (*Ch*.) *tomensis* as sister to *M*. (*Ch*.) *pumilus* and *M*. (*Ch*.) *leucogaster*. However, this relationship was not supported by the topology based on the Cytb when other related species ( *M. condylurus* and *M. leucostigma* ) were added into the analysis. Nonetheless, this gene supported the inclusion of *M*. (*Ch*.) *tomensis* within the same monophyletic group formed by the majority of the African species included within the subgenus *Chaerephon*: *M*. (*Ch*.) *atsinanana*, *M*. (*Ch*.) *pumilus*, *M*. (*Ch*.) *leucogaster* and *M*. (*Ch*.) *pusillus* with *M. leucostigma* and *M. condylurus* (Figure 4). The topologies obtained under BI, ML and MP criteria were almost identical. Both phylogenies recovered a well‐supported sister relationship between *M. condylurus* and *M. leucostigma* , with *Mops midas* more ancestral to them (Figures 3 and 4).

K2P and *p*‐distance estimates were very similar; therefore, only the K2P‐corrected distances are discussed hereafter. K2P distances ranged from 0.5% between *M*. (*Ch*.) *pumilus* and *M*. (*Ch*.) *leucogaster* to 19.1% between *M*. (*Ch*.) *tomensis* and *O. madagascariensis* (Table A4 in Appendix A). The distance between the two *M*. (*Ch*.) *tomensis* samples was 0.7% despite their belonging to distant populations. The minimum estimated distance in the Cytb between *M*. (*Ch*.) *tomensis* and any of the species compared in this study was 8.5% with *M*. (*Ch*.) *pumilus* and *M*. (*Ch*.) *leucogaster*, averaging 10.2% with all species of the genus *Mops* and 18.3% when including *Otomops* and *Tadarida* (Table A4 in Appendix A).

### 3.2 Morphological Analyses

**FIGURE 5:**
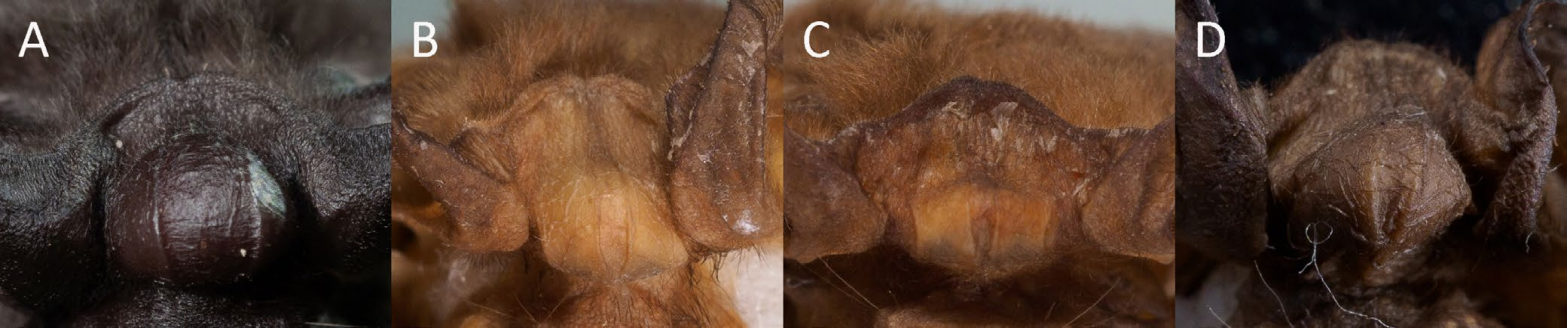
Frontal view of the interaural lobe from (A) *Mops* (*O*.) *tomensis* male (EBD 34943M), (B) *M*. (*O*.) *tomensis* female paratype (EBD 17734M), (C) *M*. (*O*.) *tomensis* female paratype (EBD 17371M), and (D) *M*. (*O*.) *gallagheri* male holotype (NHM 1976.207).

**FIGURE 6:**
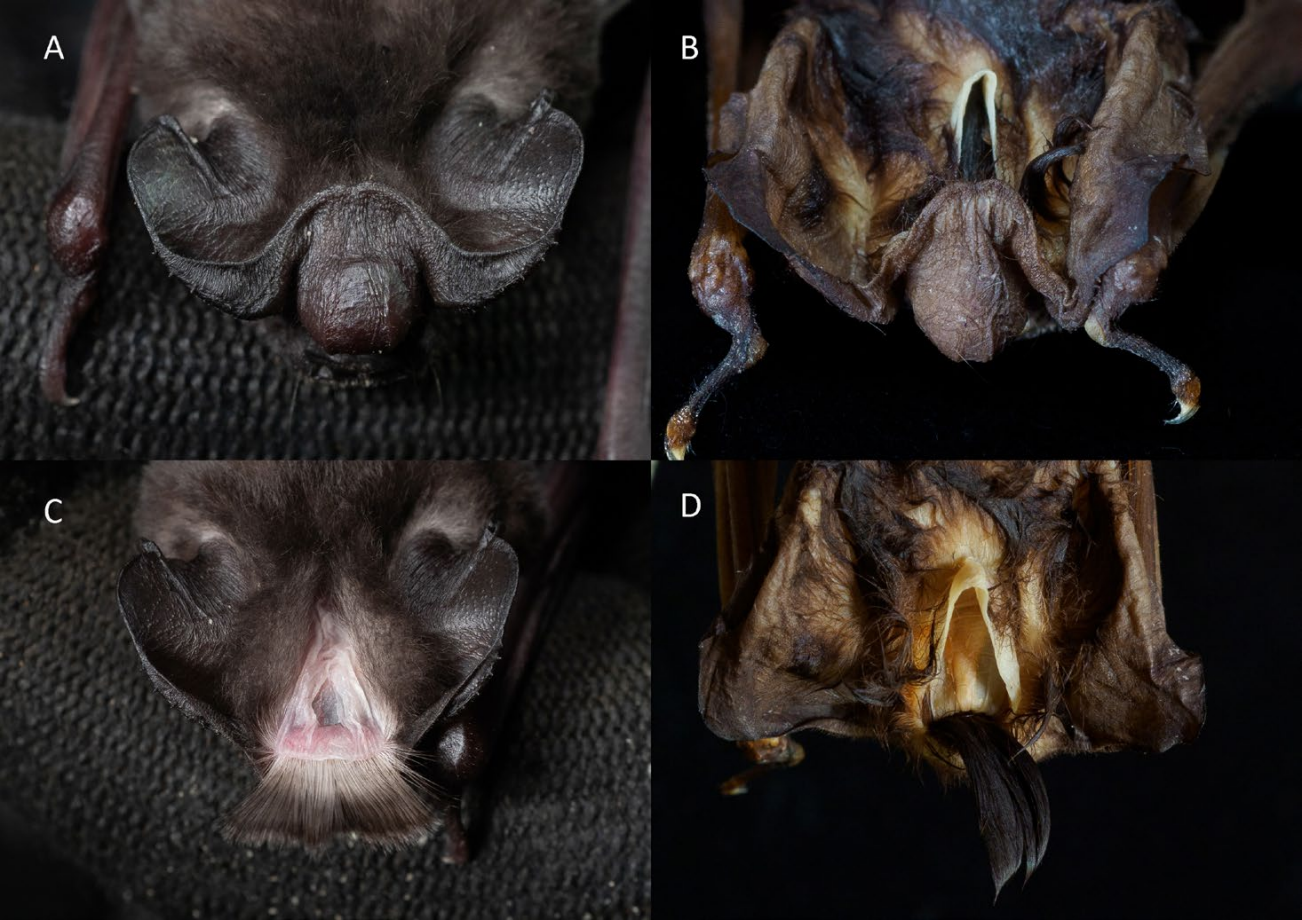
Top views of the interaural lobe close and open of (A, C) *Mops* (*O*.) *tomensis* male (EBD 34943M) and (B, D) *M*. (*O*.) *gallagheri* male holotype (NHM 1976.207).

**FIGURE 7:**
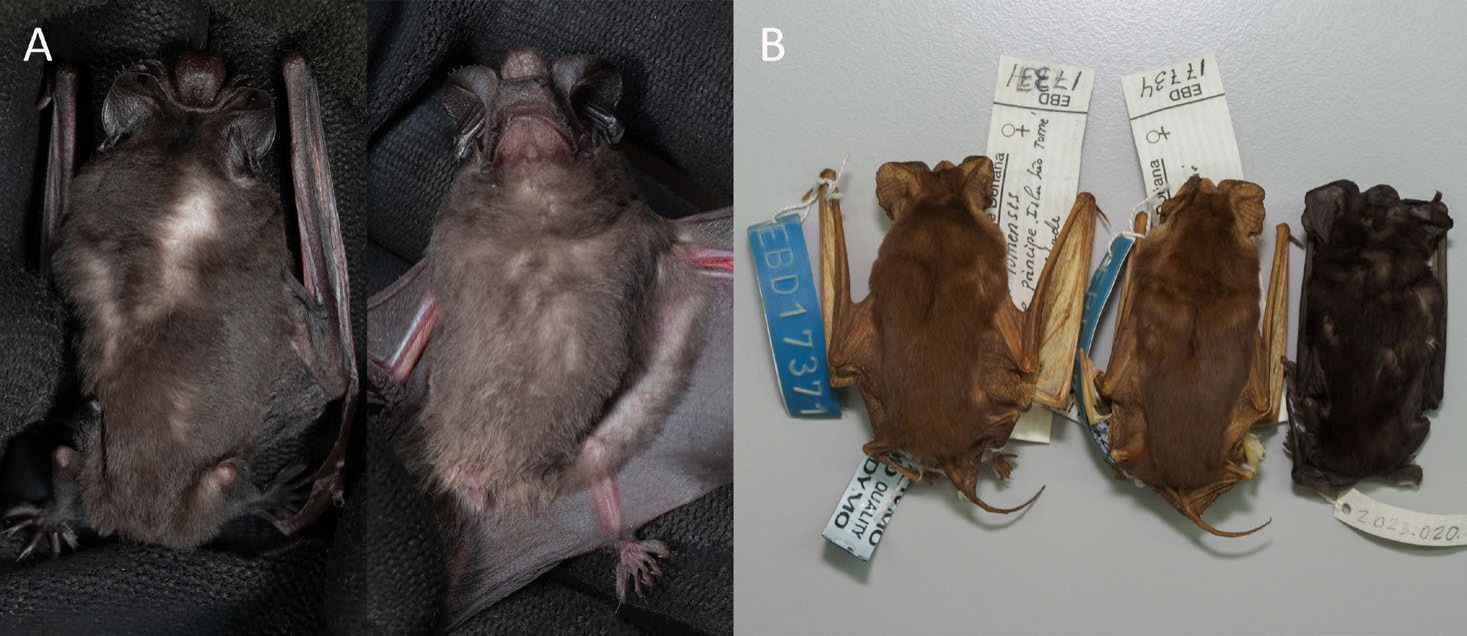
(A) Dorsal and ventral views of the pelage and skin of *Mops (O.) tomensis* male (EBD 34943M). (B) Left to right, *M. (O.) tomensis* female paratypes (EBD 17371M and EBD 17734M) and *M. (O.) tomensis* male holotype (EBD 34943M).

**FIGURE 8:**
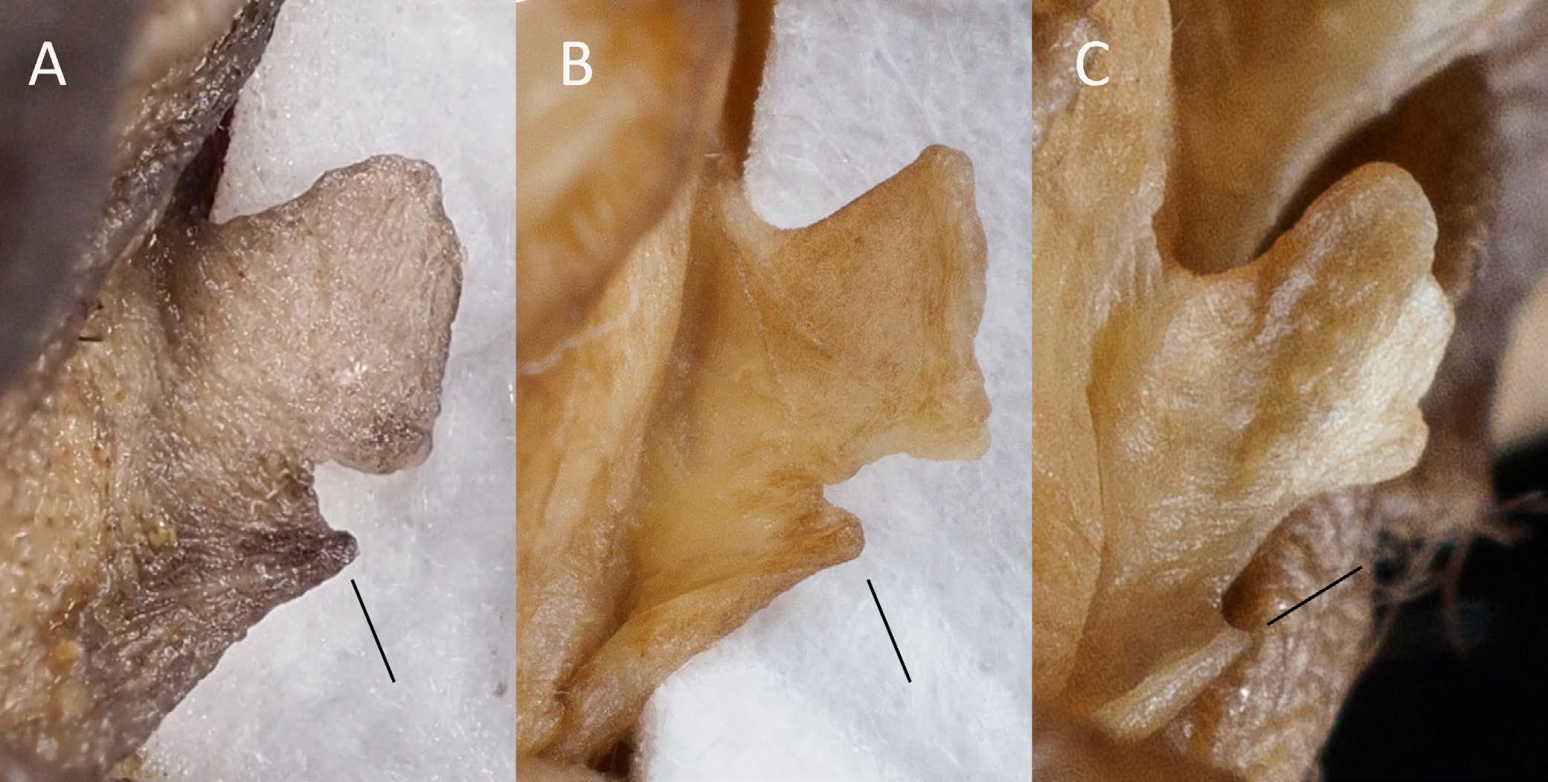
View of the left tragus from (A) *Mops* (*O*.) *tomensis* male (EBD 34943M), (B) *M*. (*O*.) *tomensis* female paratype (EBD 17371M) and (C) *M*. (*O*.) *gallagheri* male holotype (NHM 1976.207). The black line indicates the basal lobe.

**FIGURE 9:**
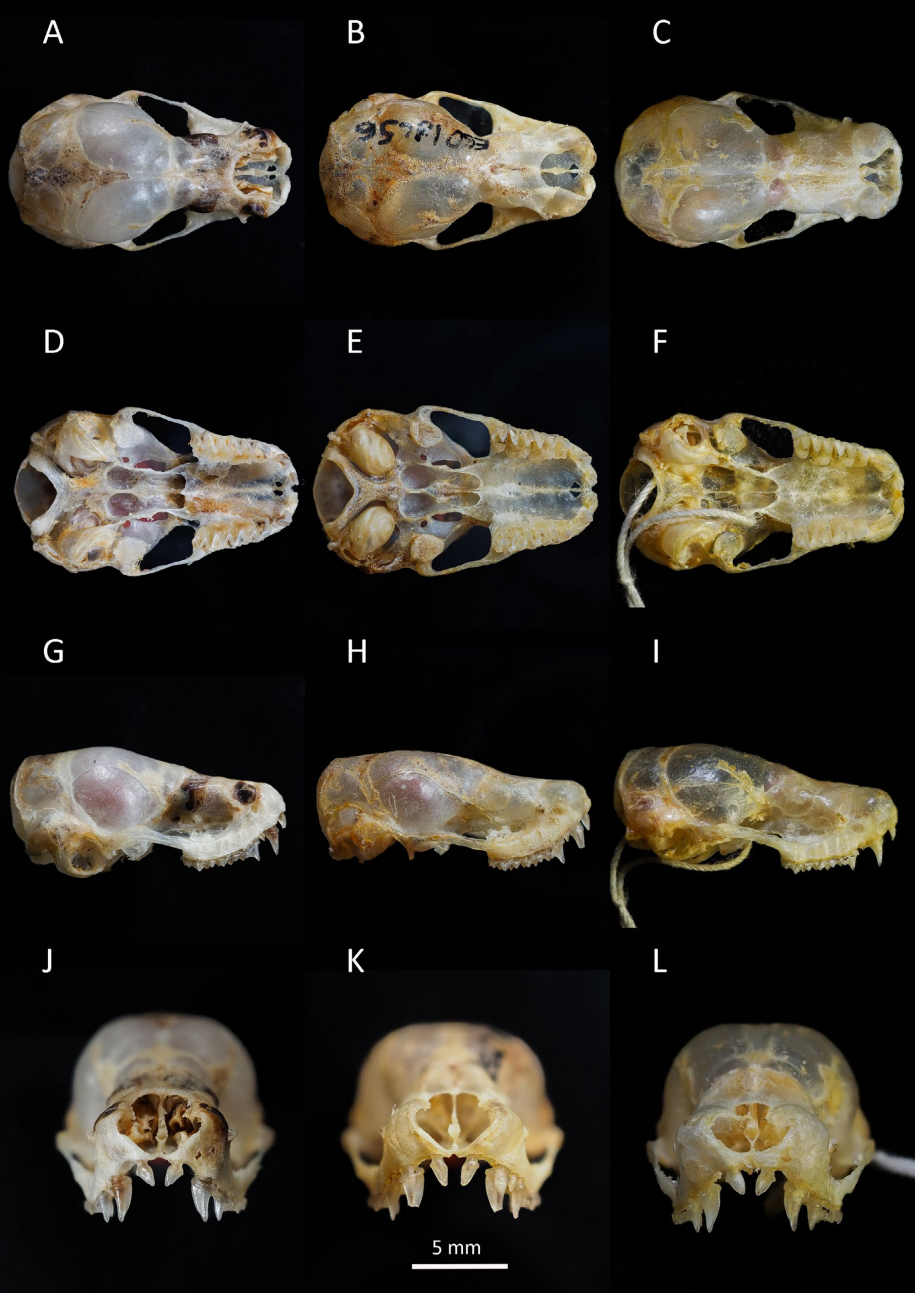
Comparison of cranial features in (A, D, G, J) *Mops (O.) tomensis* male (EBD 34943M), (B, E, H, K) *M. (O.) tomensis* female holotype (EBD 18256M) and (C, F, I, L) *M. (O.) gallagheri* male holotype (NHM 1976.207). From top to bottom: dorsal, ventral, and lateral view of the skull and frontal view of incisors.

In concordance with the molecular results, the free‐tailed bat from Piedra de Nzas also fits the distinctive morphological characteristics of *M*. (*Ch*.) *tomensis* (Juste and Ibáñez 1993) with similar measurements. Therefore, the Equatoguinean *M*. (*Ch*.) *tomensis* represents the first male known for *M*. (*Ch*.) *tomensis* as the holotype and paratypes of this species were females (Juste and Ibáñez 1993). External measurements for the *M*. (*Ch*.) *tomensis* male are as follows: total length, 75.6 mm; tail length, 30.0 mm; ear length, 14.3 mm; hind foot length, 6.8 mm; and forearm length, 36.5 mm. As differential characters, this male of *M*. (*Ch*.) *tomensis* has a prominent interaural tegumentary lobe (Figure 1), which is divided into three parts by two longitudinally septa in which the middle part is broader than the lateral ones (Figure 5A); the interaural lobe contains a pouch with a tuft of hairs of ca. 5.0 mm in length and extends slightly beyond the muzzle when unfolded (Figure 6C); the dorsal and ventral pelage are dark (Figures 1 and 7A); the tragus is slightly trilobate with the external lobes wider than the base (Figure 8A); and a prominent pair of nasal swellings surrounds the nasal aperture (Figure 9A).

Both male and female of *M*. (*Ch*.) *tomensis* share an inflated anterior rostrum of the skull with the *M*. (*Ch*.) *gallagheri* male from the Congo Basin (Figure 9), and the males of the two species share the prominent interaural lobe containing a pouch (Figure 6). So far, these bizarre features are found only in these two species along the entire Molossidae family, supporting the distinction of a new subgenus, as Harrison (1975) anticipated when describing *M*. (*Ch*.) *gallagheri*. Therefore, we formally describe herein a new subgenus based on the morphological and molecular evidence presented above.

## 4 Discussion

### 4.1 *Mops* (*Ornatomops*) Subgen. Nov.

#### 4.1.1 Type Species

*Mops Ornatomops gallagheri* Harrison (1975).

#### 4.1.2 Etymology

*Ornatomops* comes from the Latin word ‘ornatus’ meaning ‘ornament’ and *mops* is from a Malayan word that means ‘bat’.

#### 4.1.3 Other Species Included in the New Subgenus

*Mops Ornatomops tomensis* (Juste and Ibáñez 1993).

#### 4.1.4 Diagnosis

These are small‐sized free‐tailed bats of the Molossidae family with a well‐defined interaural lobe, which projects well forward over the muzzle and dominates the lateral profile of the head (Figure 1). The interaural lobe contains a pouch with a frontal tuft of hairs in males (Figure 6A,B). When displayed, this tuft comprises dark grey to brown hairs and is half the length of *M*. (*O*.) *gallagheri* (4.96 vs. 8.27 mm) (Figure 6C,D). The tragus is characteristically minute, naked and bearing three lobes ranging from weakly visible to conspicuous on its upper margin; and a pointy basal lobe (Figure 8A–C). The pelage of the upper and underparts is variably coloured, being dark grey in the male of *M*. (*O*.) *tomensis* and brown in the females of *M*. (*O*.) *tomensis* and in *M*. (*O*.) *gallagheri* (Figure 7B). Wing membranes of *M*. (*O*.) *tomensis* are translucent, whereas they are uniformly greyish and less translucent in *M*. (*O*.) *gallagheri*. Gular glands are absent in all members of this group. The cranium is slightly inflated to relatively flattish in lateral profile, with males presenting the most inflated skulls (Figure 9G–I). From a dorsal view, the cranium has a prominent pair of nasal swellings surrounding the nasal aperture of the skull (Figure 9A–C). The lacrimal bones are more developed in males than in females. The palatal emargination is small or absent (Figure 9D–F). The upper incisors are relatively pointed and directed lingually in the frontal view (Figure 9J–L). The anterior upper premolar is minute and in line with the rest of the teeth.

#### 4.1.5 Distribution

This subgenus is only known from three localities in Central Africa: São Tomé Island, mainland Equatorial Guinea and the Democratic Republic of the Congo (Harrison 1975; Juste and Ibáñez 1993) (Figure 2). The two species are associated with equatorial tropical forests.

#### 4.1.6 Systematic Relationships

The subgenus *Ornatomops* forms a clade seemingly located between *Chaerephon* and *Mops*, as currently understood. This may change once the internal evolutionary relationships of the Molossidae family are resolved, including the definitions of *Chaerephon* and *Mops*.

Few molecular studies have focused on the free‐tailed bats (F. Molossidae), and none have included all their genera. Many aspects of this group's evolutionary history remain unclear. For instance, the basal relationships within the subfamily Molossinae are still unsolved (Jones et al. 2002, 2005; Lamb et al. 2008, 2011; Agnarsson et al. 2011; Ammerman et al. 2012, 2013; Amador et al. 2018). Similarly, the situation for the African free‐tailed bats within the genera *Chaerephon*, *Mops* and *Tadarida* is deeply entangled (Lamb et al. 2011; Ammerman et al. 2012, 2013; Amador et al. 2018). Further, *M*. (*Ch*.) *bivittatus* appears as the most basal to all *Mops* taxa in a well‐supported clade suggesting that a revision and redefinition of *Chaerephon* is needed as it appears clearly polyphyletic (Figures 3 and 4). In this complex scenario, our limited molecular analysis reinforces that *M*. (*O*.) *tomensis* is an independent lineage that is apparently related to the *Mops* (*Ch*.) clade (Figures 3 and 4).

The morphological features (interaural lobe with globular swelling and inflations in the skull rostrum) shared by *M*. (*O*.) *gallagheri* and *M*. (*O*.) *tomensis* are so distinct and remarkable that they were considered to support a generic rank according to the notes of Harrison (1975) in the description of *M*. (*O*.) *gallagheri*. Nevertheless, certain similarities with the African *Mops major* and particularly with the interaural band described for the Asian *Mops* (*Ch*.) *johorensis* from the Malay Peninsula (Hill 1974), refrained Harrison from the description of the new genus. However, the African *Ornatomops* subgen. nov. presents a remarkably larger, bulbous‐shaped interaural band with respect to these two species ( *M. major* and *M*. (*Ch*.) *johorensis*) and lacks the septum that partially divides the pocket found in *M*. (*Ch*.) *johorensis* (Roslan et al. 2016) (Figure 6A,B). Moreover, the interaural band in *M*. (*Ch*.) *johorensis* does not project forward covering the muzzle as with *Ornatomops* subgen. nov. species (Figures 1 and 6C,D). Additionally, the nasal inflation of the skull, so characteristic of the *Ornatomops* subgen. nov., is lacking in these two species, which also show smaller narial emarginations (Figure 9A–C).

The partial reconstructed Cytb sequence of one of the paratypes of *M*. (*O*.) *tomensis*, preserved in formalin, is supporting the distinction of this new taxon. The genetic distance with other closely related lineages, along with its position between *Mops* and several African *Chaerephon* in the phylogenetic reconstructions, strongly supports a subgeneric rank for this new lineage. It is tentatively included within *Mops* (*sensu* Napier 2013). The genetic distance observed in the mtDNA Cytb is similar between *Mops* and *Chaerephon*, at approximately 10%. In the Neotropics, similar genetic distances (9.7%) have been reported for Cytb in *Cynomops* and of 12.1% for Cytochrome c Oxidase subunit I (COI) in *Eumops* (Moras et al. 2016; López‐Baucells et al. 2018). Unfortunately, DNA sequences of *M*. (*O*.) *gallagheri* are not available at present as it is known just from the old holotype, but its morphological characters clearly support its inclusion in the new subgenus. Our topologies suggest that, within the currently limited phylogenetic framework of the group, the new subgenus should be provisionally included within the genus *Mops*. It appears basal to a well‐defined clade of closely related African *Chaerephon* species (e.g., *M. (Ch.) atsinanana*, *M. (Ch.) leucogaster*, *M. (Ch.) pumilus* and *M. (Ch.) pusillus*) and the group comprising *M. condylurus* and *M. leucostigma* (Figure 4). Although the systematic placement of *Ornatomops* subgen. nov. remains dependent on a comprehensive molecular revision of these lineages, the advantage of defining this subsidiary rank (*sensu* Dubois 2007) is its ability to categorise the two closely related species into a morphologically diagnosable clade. This approach allows for taxonomic clarity and acceptance without deviating from the conventional binomial nomenclature (Teta 2019). Besides, the recognition of *Ornatomops* subgen. nov. does not bring additional instability or confusion to this still unresolved group of bats.

As is the case with the molecular data, several morphological characters support a closer relationship of *Ornatomops* subgenus with *Chaerephon* rather than with *Mops* (Freeman 1981; Legendre 1984; Gregorin and Cirranello 2016), namely, the small or absent palatal emargination; the separated, frontally piriform and laterally triangular upper incisors; the anterior upper premolar smaller than in *Mops*; a M^3^ third ridge equal to or longer than the second ridge; the lower canines almost in contact; and the small coronoid process and less developed lambdoidal crest.

Several bat families show reverse sexual dimorphism (RSD), where females tend to be larger than males (Ralls 1976; Lindenfors et al. 2007; Racey 2009). RSD is well documented across Emballonuridae, Noctilionidae, Phyllostomidae and Vespertilionidae (Meyers 1978; Stevens and Platt 2015; Adams et al. 2020; Ospina‐Garcés and León‐Paniagua 2021). RSD can result from evolutionary pressures by different factors, including reproduction and flight energetics (Meyers 1978). In contrast, free‐tailed bats commonly exhibit typical mammal size dimorphism towards larger males (Freeman 1981; Ober et al. 2017; Boonpha et al. 2019). Some African free‐tailed bats exhibit typically more robust males that present pronounced morphological features likely playing an active role in sexual selection. For instance, *M. thersites* males display conspicuous canines and well‐developed cranial crests, and *M*. (*Ch*.) *chapini* males are characterised by a prominent interaural crest (Freeman 1981; Happold 2013b; Happold and Cotterill 2013). Interestingly, a RSD seems to apply again within the new subgenus *Ornatomops*, as female *M*. (*O*.) *tomensis* are slightly larger than both male *M*. (*O*.) *tomensis* and *M*. (*O*.) *gallagheri* and also show a more robust dentition (Figure 9G–L, Table A5 in Appendix A). Different dimorphism modes within the same family are also found in other bats like Hipposideridae or Rhinonycteridae. Sexual dimorphism can also be manifested in the pelage colouration (Racey 2009). The male of *M*. (*O*.) *tomensis* shows a distinctive darker colouration compared to the light brown of the females (Figure 7B). A similar pattern has been observed in the Malagasy species *Tadarida fulminans* , where males display a darker reddish‐brown dorsal colouration and a yellowish‐pink ventral side, while females and subadults exhibit a chocolate‐brown dorsal colouration with a whitish ventral side (Taylor et al. 2019). We still need to study more specimens to assess completely the sexual dimorphism within the *Ornatomops* subgenus, including within *M*. (*O*.) *gallagheri*. In fact, we must consider that some differences found in external and skull characteristics between *M*. (*O*.) *tomensis* females from São Tomé Island and the male collected in mainland Equatorial Guinea could originate from the geographic isolation of the two populations instead of from sexual selection. Nonetheless, the lack of genetic differentiation between these two populations is quite remarkable, keeping in mind that the small oceanic island of São Tomé is found more than 300 km offshore and from which *M*. (*O*.) *tomensis* was—so far—considered endemic like many other animals and plants found on the island (e.g., Rainho et al. 2022; Stévart et al. 2022). This insular population is situated over 500 km from the continental locality where the male was collected (Piedra de Nzas forest) (Figure 2). Despite this, only one mutation was identified along the several‐hundred‐bp‐long fragment of the Cytb gene used for our molecular comparison. However, it is crucial to analyse a larger number of genes, both mitochondrial and nuclear, to determine whether the observed genetic similarity reflects gene flow between the island and continental populations. Additionally, further investigation into the species' natural history and ecology is needed to explain this remarkable finding.

The functionality of the unique morphological features that characterise the new subgenus is still to be understood. The interaural lobe with a swelling pocket, encasing a tuft of hairs, presented in *M*. (*O*.) *tomensis* and *M*. (*O*.) *gallagheri* males is similar to the one presented by *M*. (*Ch*.) *chapini* males. In *M*. (*Ch*.) *chapini*, this is expected to be related to the release of hormones or other chemicals during specific courtship displays. Thus, we expect it to be used for similar purposes in *M*. (*O*.) *tomensis* and *M*. (*O*.) *gallagheri* males. On the other hand, Harrison (1975) speculates on the possible role played by the inflations in the rostrum in the echolocation emissions of this bat. Nevertheless, the only recordings assigned to *M*. (*O*.) *tomensis*—the sister species of *M*. (*O*.) *gallagheri* that present also inflations—seem to show no peculiarities in comparison to the calls of the similar species *M*. (*Ch*.) *pumilus* (without inflations) apart from being slightly lower in maximum frequency (Rainho et al. 2022).

Finally, Central Africa is experiencing a high rate of habitat transformation. Accurate and up‐to‐date information on rare bat species is urgent. This data are essential for re‐evaluating their taxonomic classification and implementing effective conservation measures to preserve them. Systematic surveys have already proved useful for bat conservation in Central Africa. A good example is the rediscovery of the critically endangered *Rhinolophus hilli* in Uganda 40 years after the last reported observation (Flanders et al. 2022). Both species, *M*. (*O*.) *tomensis* and *M*. (*O*.) *gallagheri*, were caught in rainforest and we can infer a forest dependency within the Congolian rainforest, which is becoming increasingly endangered by human activities (Potapov et al. 2017, 2022). Indeed, *M*. (*O*.) *tomensis* is considered endangered (Rainho et al. 2022) and *M*. (*O*.) *gallagheri* still needs to be assessed by the IUCN.

## Author Contributions

**Laura Torrent:** conceptualization (equal), data curation (lead), formal analysis (equal), funding acquisition (lead), methodology (equal), project administration (lead), writing – original draft (lead). **Inazio Garin:** investigation (equal), writing – review and editing (equal). **Joxerra Aihartza:** investigation (supporting), writing – review and editing (supporting). **Aline Méndez‐Rodríguez:** data curation (supporting), formal analysis (supporting), methodology (supporting), writing – original draft (supporting). **Esther Abeme Nguema Alene:** investigation (supporting), methodology (equal), project administration (supporting), resources (supporting). **Javier Juste:** conceptualization (lead), formal analysis (equal), funding acquisition (supporting), investigation (equal), methodology (equal), project administration (supporting), resources (supporting), supervision (lead), writing – original draft (equal).

## Conflicts of Interest

The authors declare no conflicts of interest.

## Data Availability

The Cytb, NADH1 and RAG2 sequence data generated from this study are available publicly at https://www.ncbi.nlm.nih.gov/nucleotide/ under the GenBank accession numbers PV366758 to PV366767.

## Appendix A

**TABLE A1:** Sets of primers used to amplify each molecular marker used in this study (Cytb, NADH1 and RAG2).

Markers	Primers	Sequence	Source
Cytb	L‐14724	5′‐CGAAGCTTGATATGAAAAACCATCGTTG‐3′	Irwin et al. (1991)
	MVZ‐16	5′‐AAATAGGAARTATCAYTCTGGTTTRAT‐3′	Smith and Patton (1993)
	MOLCIT‐F	5′‐AATGACATGAAAAATCACCGTTGT‐3′	Ibáñez et al. (2006)
NADH1	ER66ND1	5′‐GTATGGGCCCGATAGCTT‐3′	Mayer and von Helversen (2001)
	ND1‐F2	5′‐GGCAGAGACCGGTAATTGCATAA‐3′	Kawai et al. (2002)
RAG2	F1	5′‐GGCTGGCCCAARAGATCCTG‐3′	Baker et al. (2000)
	R1	5′‐AACYTGYTTATTGTCTCCTGGTATGC‐3′	Baker et al. (2000)
	F2	5′‐TTTGTTATTGTTGGTGGCTATCAG‐3′	Baker et al. (2000)

**TABLE A2:** Molossid bats of the genera *Mops*, *Otomops* and *Tadarida* used in the molecular analyses with Cytb marker, along with their associated GenBank accession numbers, country of origin, voucher number and references.

Species	Cytb GenBank number	Country	Voucher	References
*Mops* (*Ch*.) *atsinanana* (Cat1MA)	JN867845	Madagascar	FMNH 184655	Lamb et al. (2012)
*Mops* (*Ch*.) *atsinanana* (Cat2MA)	KF193644	Madagascar	FMNH 187931	Naidoo et al. unpublished
*Mops* (*Ch*.) *atsinanana* (Cat3MA)	KF193642	Madagascar	UADBA 43914	Naidoo et al. unpublished
*Mops* (*Ch*.) *bivittatus* (CbiKEN)	MN064727	Kenya	FMNH 234215	Lutz et al. (2019)
*Mops condylurus* (Mco1RC)	MZ265567	Republic of the Congo	COG 0535	Seifert et al. (2022)
*Mops condylurus* (Mco2KE)	MK330941	Kenya	B 241	Forbes et al. (2019)
*Mops condylurus* (Mco3AF)	GQ489181	Togo	FMNH 265	Naidoo et al. unpublished
*Mops* (*Ch*.) *leucogaster* (Cle1CO)	EU716041	Mayotte Island, Comoro	FMNH 194019	Goodman et al. (2010)
*Mops* (*Ch*.) *leucogaster* (Cle2CO)	GQ489167	Madagascar	FMNH 185020	Goodman et al. (2010)
*Mops leucostigma* (Mlu1MA)	FJ5462561	Comoro Islands	FMNH 194503	Hoosen et al. unpublished
*Mops leucostigma* (Mlu2MA)	FJ546257	Comoro Islands	FMNH 194505	Hoosen et al. unpublished
*Mops leucostigma* (Mlu3MA)	GQ489180	Madagascar	FMNH 264	Naidoo et al. unpublished
*Mops midas* (Mmi1AF)	EF474049	Madagascar	FMNH 185157	Ratrimomanarivo et al. (2007)
*Mops midas* (Mmi2AF)	EF474048	Madagascar	FMNH 185156	Ratrimomanarivo et al. (2007)
*Mops midas* (Mmi3AF)	EF474047	Madagascar	FMNH 185155	Ratrimomanarivo et al. (2007)
*Mops* (*Ch*.) *pumilus* (Cpm3DR)	MZ265613	Republic of the Congo	COG 0541	Seifert et al. (2022)
*Mops* (*Ch*.) *pumilus* (Cpm4RC)	MZ265611	Republic of the Congo	COG 0539	Seifert et al. (2022)
*Mops* (*Ch*.) *pumilus* (CpmEG1)	PV366760	Oyala, Equatorial Guinea	—	This study
*Mops* (*Ch*.) *pumilus* (CpmEG2)	PV366761	Ncama, Equatorial Guinea	—	This study
*Mops* (*Ch*.) *pumilus* (CpmST1)	PV366762	São Tomé Island	EBD 22215M	This study
*Mops* (*Ch*.) *pumilus* (CpmST2)	PV366763	São Tomé Island	EBD 22218M	This study
*Mops* (*Ch*.) *pumilus* (CpmST3)	PV366764	São Tomé Island	EBD 22219M	This study
*Mops* (*Ch*.) *pumilus* (CpmST4)	PV366765	São Tomé Island	EBD 22220M	This study
*Mops* (*Ch*.) *pusillus* (Cps1CO)	GQ489155	Comoro Islands	SMG 16315	Naidoo et al. unpublished
*Otomops harrisoni* (Oha1EA)	MH010749	Rwanda	PWW 3273	Patterson et al. (2018)
*Otomops harrisoni* (Oha2KE)	MH010747	Kenya	FMNH‐NMK 187140	Patterson et al. (2018)
*Otomops harrisoni* (Oha3KE)	MH010742	Kenya	FMNH 225590	Patterson et al. (2018)
*Otomops madagascariensis* (Oma2MA)	MH010750	Madagascar	FMNH 177398	Patterson et al. (2018)
*Otomops madagascariensis* (Oma3MA)	KR606335	Madagascar	UADBA 32330	Gomard et al. (2016)
*Otomops madagascariensis* (Oma4MA)	KJ433741	Madagascar	SMG 16459f	Lamb et al. (2008)
*Otomops martiensseni* (Omr1AF)	MH010753	Rwanda	PWW 3271	Patterson et al. (2018)
*Otomops martiensseni* (Omr3KE)	AY591532	South Africa	—	Rahman et al. unpublished
*Tadarida insignis* (Tin1AS)	MG570074	China	YN‐63	Huang et al. unpublished
*Mops* (*O*.) *tomensis* (Msp1EG)	PV366758	Piedra de Nzas, Equatorial Guinea	EBD 34943M	This study
*Mops* (*O*.) *tomensis* (Mtom1)	PV366759	São Tomé Island	EBD 17371M	This study

*Note:* The acronyms of each individual are provided in brackets.

**TABLE A3:** Molossid bats of the genera *Mops*, *Otomops* and *Tadarida* used in the molecular analysis with the concatenated Cytb, NADH1 and RAG2 markers, along with their associated GenBank accession numbers, country of origin, voucher number and references.

Species	GenBank number			Country	Voucher	References
	Cytb	NADH1	RAG2			
*Mops condylurus* (Mco56A)	HM802913	HQ671540	HQ671589	South Africa	DSJ 63 (NADH1, RAG2) DM 6332 (Cytb)	Ammerman et al. (2012)
*Mops* (*Ch*.) *jobimena* (Cjo23M)	HM802932	HQ671527	HM631627	Madagascar	FMNH 176329 (NADH1) FMNH 175992 (Cytb, RAG2)	Lamb et al. (2011), Ammerman et al. (2012)
*Mops* (*Ch*.) *leucogaster* (Cle78M)	HM802901	HQ671525	HQ671582	Madagascar	FMNH 176330 (NADH1, RAG2) HM 802901 (Cytb)	Lamb et al. (2011), Ammerman et al. (2012)
*Mops leucostigma* (Mlu56M)	HQ384484	HQ671541	HQ671592	Madagascar	FMNH 176041 (NADH1, RAG2) FMNH 188009 (Cytb)	Lamb et al. (2011), Ammerman et al. (2012)
*Mops* (*Ch*.) *pumilus* (Cpm41C)	MZ265611	HQ671526	GU328051	Uganda (NADH1, RAG) Republic of the Congo (Cytb)	FMNH 137634 (NADH1, RAG2) COG 0539 (Cytb)	Lack et al. (2010), Ammerman et al. (2012), Seifert et al. (2022)
*Mormopterus jugularis* (Mju45M)	HM802919	HQ671543	HM631655	Madagascar	FMNH 176341 (NADH1) FMNH 184347 (Cytb, RAG2)	Lamb et al. (2011), Ammerman et al. (2013)
*Otomops madagascariensis* (Oma67M)	HQ384485	HQ384490	HQ671550	Madagascar	FMNH 176354 (NADH1) UADBA SMG 10996 (Cytb, RAG2)	Lamb et al. (2011), Ammerman et al. (2013)
*Sauromys petrophilus* (Spe23A)	HM802931	HQ671553	HQ671598	South Africa	CM 105758 (NADH1, RAG2) DM 8613 (Cytb)	Ammerman et al. (2013)
*Tadarida brasiliensis* (Tbr23A)	L19734	HQ671554	HQ671599	Argentina	OMNH 23758 (NADH1, RAG2) LSUMZ 23909 (Cytb)	Sudman et al. (1994), Ammerman et al. (2013)
*Mops* (*O*.) *tomensis* (Msp1EG)	PV366758	PV366766	PV366767	Piedra de Nzas, Equatorial Guinea	EBD 34943M	This study

**TABLE A4:** Genetic distances (bottom—Kimura 2‐parameter [K2P] and top—*p*‐distance), based on a 623 bp fragment of Cytb, among the species studied within the genus *Mops*, *Otomops* and *Tadarida*.

	Mtom	Cpum	Cleo	Cpus	Cats	Mluc	Mcon	Mmid	Cbiv	Ohar	Omrt	Omad	Tins
*Mops* (*O*.) *tomensis* (Mtom)		7.9%	7.9%	8.2%	9.2%	9.4%	10.1%	10.2%	11.9%	15.8%	15.7%	16.7%	16.1%
*Mops* (*Ch*.) *pumilus* (Cpum)	8.5%		0.5%	1.5%	1.7%	8.5%	8.6%	9.9%	10.6%	14.7%	15.3%	15.1%	14.6%
*Mops* (*Ch*.) *leucogaster* (Cleo)	8.5%	0.5%		1.2%	1.8%	8.3%	8.3%	9.5%	10.9%	14.0%	14.4%	14.5%	13.5%
*Mops* (*Ch*.) *pusillus* (Cpus)	8.9%	1.5%	1.2%		2.9%	8.6%	8.5%	10.2%	10.8%	14.4%	14.8%	14.6%	14.0%
*Mops* (*Ch*.) *atsinanana* (Cats)	10.0%	1.7%	1.9%	3.0%		8.7%	8.7%	10.1%	11.1%	13.9%	14.2%	14.3%	13.5%
*Mops leucostigma* (Mluc)	10.3%	9.2%	8.9%	9.3%	9.4%		2.3%	10.7%	10.1%	15.3%	15.4%	14.9%	16.0%
*Mops condylurus* (Mcon)	11.1%	9.4%	9.0%	9.1%	9.4%	2.4%		10.5%	9.9%	15.2%	15.4%	15.2%	15.5%
*Mops midas* (Mmid)	11.2%	10.9%	10.4%	11.2%	11.1%	11.8%	11.5%		10.8%	14.9%	14.8%	15.3%	13.7%
*Mops* (*Ch*.) *bivittatus* (Cbiv)	13.2%	11.7%	12.1%	11.9%	12.3%	11.0%	10.8%	12.0%		12.9%	13.1%	13.2%	13.7%
*Otomops harrisoni* (Ohar)	18.0%	16.7%	15.7%	16.2%	15.6%	17.4%	17.2%	16.8%	14.3%		2.5%	3.3%	12.3%
*Otomops martiensseni* (Omrt)	17.8%	17.4%	16.2%	16.7%	15.9%	17.4%	17.4%	16.6%	14.5%	2.6%		4.0%	13.4%
*Otomops madagascariensis* (Omad)	19.1%	17.0%	16.3%	16.4%	16.1%	16.8%	17.2%	17.4%	14.7%	3.4%	4.2%		12.9%
*Tadarida insignis* (Tins)	18.4%	16.4%	15.1%	15.7%	15.1%	18.3%	17.7%	15.4%	15.4%	13.6%	14.9%	14.3%	

**TABLE A5:** Measurements (mm) of *Mops tomensis* male and comparison species (*Mops (O.) gallagheri, Mops (O.) tomensis* and *Mops (Ch.) chapini*).

Species	*Mops* (*O*.) *tomensis* (male)	*Mops* (*O*.) *tomensis* (holotype)	*Mops* (*O*.) *tomensis* (paratype)	*Mops* (*O*.) *tomensis* (paratype)	*Mops* (*O*.) *gallagheri* (holotype)	*Mops (Ch.) chapini* (holotype)
Collection number	EBD 34943M	EBD 18256M	EBD 17734M	EBD 17371M	NHM 1976.207	NHM 1937.12.8.25
GLS	15.8	15.5	15.2	16.0	15.9	17.1
ZW	9.1	9.1	9.1—broken	9.1	8.9	10.4
MW	8.8	8.8	8.4	8.5	8.7	9.4
GHS	7.5	6.1	6.9	7.4	6.4	6.7
CbL	14.6	14.2	13.7	14.0	14.4	15.5
IOW	4.0	3.8	3.9	3.9	4.1	3.9
pal	6.0	6.2	6.7	6.6	6.7	7.1
PL	5.8	6.0	6.3	6.5	6.7	7.1
I‐I	0.7	0.9	0.9	0.9	0.7	0.8
C‐C	3.3	3.5	3.5	3.4	3.8	4.1
C‐M^3^	5.3	5.5	5.7	5.5	5.7	5.9
M^3^‐M^3^	6.5	6.9	6.9	6.7	6.1	7.4
FA	36.5	38.5	35.5	38.0	37.6	39.0
W	7.3	7.2^a^	8.9	9.0	—	—
TL	75.6	92.0	77.0	90.0	77.0	—
Sex	M	F	F	F	M	M

*Note:* See Section 2 for variable abbreviations.

^a^ Measurement for *M.(O.) tomensis* holotype weight after (Juste and Ibáñez 1993).
